# Dopamine D1 receptor-mediated activation of the ERK signaling pathway is involved in the osteogenic differentiation of bone mesenchymal stem cells

**DOI:** 10.1186/s13287-019-1529-x

**Published:** 2020-01-03

**Authors:** Chen-Xi Wang, Xi-Yuan Ge, Ming-Yue Wang, Ting Ma, Yu Zhang, Ye Lin

**Affiliations:** 1 Department of Implantology, Peking University School and Hospital of Stomatology & National Clinical Research Center for Oral Diseases & National Engineering Laboratory for Digital and Material Technology of Stomatology & Beijing Key Laboratory of Digital Stomatology, Beijing, 100081 People’s Republic of China; 2 Central Laboratory, Peking University School and Hospital of Stomatology, Beijing, 100081 People’s Republic of China

**Keywords:** Dopamine, D1 receptor, Bone mesenchymal stem cells, Cellular differentiation, ERK signaling pathway

## Abstract

**Background:**

Osteogenic differentiation of bone mesenchymal stem cells (BMSCs) is regulated by numerous signaling pathways. Dopamine (DA), a neurotransmitter, has previously been demonstrated to induce new bone formation by stimulating the receptors on BMSCs, but the essential mediators of DA-induced osteogenic signaling remain unclear.

**Methods:**

In this work, we evaluated the influence of both dopamine D1 and D2 receptor activation on BMSC osteogenic differentiation. Gene and protein expression of osteogenic-related markers were tested. The direct binding of transcriptional factor, Runx2, to those markers was also investigated. Additionally, cellular differentiation-associated signaling pathways were evaluated.

**Results:**

We showed that the expression level of the D1 receptor on BMSCs increased during osteogenic differentiation. A D1 receptor agonist, similar to DA, induced the osteogenic differentiation of BMSCs, and this phenomenon was effectively inhibited by a D1 receptor antagonist or by D1 receptor knockdown. Furthermore, the suppression of protein kinase A (PKA), an important kinase downstream of the D1 receptor, successfully inhibited DA-induced BMSC osteogenic differentiation and decreased the phosphorylation of ERK1/2. Compared with P38, MAPK, and JNK, DA mainly induced the phosphorylation of ERK1/2 and led to the upregulation of Runx2 transcriptional activity, thus facilitating BMSC osteogenic differentiation. On the other hand, an ERK1/2 inhibitor could reverse these effects.

**Conclusions:**

Taken together, these results suggest that ERK signaling may play an essential role in coordinating the DA-induced osteogenic differentiation of BMSCs by D1 receptor activation.

## Background

Bone is constantly remodeled through the synchronized and balanced activities of osteoclasts and osteoblasts. This process is highly controlled by autocrine, paracrine, and endocrine factors from the external environment to ensure the systemic balance of calcium–phosphate metabolism while maintaining bone homeostasis. Previous research has reported that damaged or missing sympathetic nerves, which enter bone marrow spaces, result in abnormal bone homeostasis. Although their function within the marrow space is unclear, recent data suggest that disrupting the hypothalamic–pituitary–gonadal axis, sympathetic nervous system (SNS) stimulation and direct interaction with receptors on cell membranes may contribute to this bone loss [1]. Considering that some of the nerve terminals are in direct contact with bone cells without synapses, neurotransmitters could spillover from nerves, and several specific receptors for these neurotransmitters have previously been found on both osteoclasts and osteoblasts, such as the β2-adrenergic receptor (β2AR) [2–4]. Thus, our team focused on the interaction between neurotransmitters and their receptors.

G protein-coupled receptors (GPCRs), such as dopamine receptor (DAR), parathyroid hormone receptor (PTHR), β2AR, calcium-sensing receptor (CaSR), and 5-hydroxytryptamine receptor (5-HTR), are cell membrane proteins with a seven-transmembrane structure that triggers signals within the cells, activates or inhibits specific effectors to induce cellular responses, and regulates many functions. Approximately 30–40% of marketed drugs target these kinds of receptors, including those used to treat respiratory, cardiovascular, and central nervous system (CNS) disorders [5]. With the increasing use of antipsychotics (APs) targeting at GPCRs, its side effects on bone metabolism for both children and adults have received most of the attention [6–9]. GPCRs are also expressed within osteoblasts and osteoclasts, which are thought to be two opposite sides of a coin, and play a crucial role in modulating bone turnover, thus highlighting the potential for these receptors in the treatment of bone-related diseases, namely, osteoporosis, through a long-lasting enhancement of bone formation with the relative inhibition of bone resorption [10].

Dopamine (DA), a neurotransmitter, mediates many physiological functions, such as voluntary movement, reward, sleep regulation, feeding, affect, attention, cognitive function, olfaction, vision, hormonal regulation, and sympathetic regulation, and its deficiency or excess causes neurological and psychiatric disorders, such as Parkinson’s disease or schizophrenia. Individuals with both of these diseases have a higher risk of osteoporosis fracture than the general population, which indicates that the concentration of DA may influence bone mass [7–9]. Knockout of the dopamine transporter (DAT), which controls the activity of released DA, has been reported to reduce bone mass in mice [11, 12]. A recent study reported that risperidone (RIS), a DA receptor antagonist, could cause additional bone loss in ovariectomized (OVX) mice, which indicated that a disruption in the hypothalamic–pituitary–gonadal axis could not sufficiently explain the function of DA [13]. Several studies have reported that DA could interact with the receptors on osteoclasts to inhibit bone absorption via the NFATc-1 and c-Fos signaling pathway [14, 15]. During bone formation, bone marrow mesenchymal stem cells (BMSCs), which can self-renew and differentiate into multiple lineages of mesenchymal tissues, including the bone, cartilage, fat, muscle, and tendon, give rise to osteoprogenitor cells that then differentiate into mature osteoblasts [16–19]. Previous research has demonstrated that DA could affect BMSC proliferation and osteogenic differentiation via its receptors [13, 20, 21]. To date, five receptors of DA, D1R, D2R, D3R, D4R, and D5R, have been discovered on BMSCs [21, 22]. However, the underlying mechanism of DA-induced BMSC osteogenic differentiation remains unclear.

According to pharmacology and the ability to regulate cyclic adenosine monophosphate (cAMP) concentration, DA receptors could be divided into D1-like and D2-like subfamilies. D1R and D5R, which upregulate the concentration of cAMP, are included in the D1-like family. D2R, D3R, and D4R are included in the D2-like subfamily, and these receptors inhibit the production of cAMP by inhibiting adenylate cyclase [23, 24]. Mitogen-activated protein kinases (MAPKs) are a family consisting of a series of conserved serine/threonine protein kinases that contribute to a variety of cellular activities, such as proliferation, differentiation, apoptosis, migration, stress response, and senescence [25–27]. Typical MAPK members include extracellular signal-regulated kinase 1/2 (ERK1/2), c-Jun N-terminal kinases 1-3 (JNK1-3), and p38 isoforms (p38α, β, γ, and δ), which have previously been reported to be controlled by the concentration of cAMP. In addition, several studies have revealed MAPKs as key factors in the regulation of osteoblast cell line commitment and differentiation by enhancing the activity of Runx2 (a crucial transcription factor for osteoblast differentiation) [28–30]. Taken together, these results suggest that MAPKs may play an important role in DA-induced BMSC proliferation and differentiation.

In this study, we aimed to characterize the effects of different DA receptors on BMSCs and the possible molecular mechanism involved. Our hypothesis was that D1 receptors might upregulate the intercellular concentration of cAMP and thus activate the ERK signaling pathway, thereby enhancing the ability of Runx2 to bind to the promoters of relevant osteogenesis genes.

## Materials and methods

### BMSC culture

Human BMSCs (hBMSC passage 2) were isolated from one adult donor and purchased from Cyagen Biosciences Technology (Guangzhou, China). Rat BMSCs (rBMSC passage 2) were isolated from Sprague–Dawley rats and purchased from Cyagen Biosciences Technology (Guangzhou, China). The cells were cultured according to the manufacturer’s instructions. Briefly, the BMSCs were cultured in α-Minimal Essential Media (α-MEM; Gibco, USA) containing 10% fetal bovine serum (FBS; Gibco, USA) and 1% penicillin/streptomycin at 37 °C in a humidified atmosphere of 95% air and 5% CO_2_ with a culture medium change every 2–3 days [22].

### BMSC proliferation

BMSCs were plated onto 96-well dishes at a density of 15,000 cells per ml. After 24 h, the cells were divided in triplicate into eight groups and incubated with DA at concentrations of 0 nmol/ml, 0.5 nmol/ml, 5 nmol/ml, 50 nmol/ml, 500 nmol/ml, 5 μmol/ml, 50 μmol/ml, and 500 μmol/ml. After 1, 3, 5, and 7 days, the culture medium in each well was replaced with 150 μl 10% CCK-8 buffer (CCK-8, Dojindo, Japan) and incubated at 37 °C for another 2 h. The optical density (OD) of each well was recorded on an ELX-808 Absorbance Microplate Recorder (BioTek, Winooski, VT) at 450 nm. The mean of each triplicate reading was used for the analysis, and the experiment was repeated three times independently.

### BMSC osteogenic differentiation

For osteogenic differentiation, BMSCs were seeded onto a 6-well plate at a density of 40,000 cells per ml and a 12-well plate at 20000 cells per ml. When the cells reached 80–90% confluence, the culture medium was replaced with osteogenic medium (OriCell™ Human MSC Osteogenic Differentiation Medium, Cyagen Biosciences, Guangzhou, China), and the media were changed after 3–4 days. After 14 days, osteogenic differentiation was evaluated by ARS staining (Cyagen Biosciences, Guangzhou, China). For the quantification analysis, the stained disks were desorbed using 10% cetylpyridinium chloride (Sigma, USA). The absorbance values at 590 nm were recorded. The total protein content, which was determined by a bicinchoninic acid (BCA) protein assay kit (Thermo, USA), was used for normalization.

### Alkaline phosphatase (ALP) activity assay and ALP staining

To evaluate the ALP activity, BMSCs were seeded onto a 12-well plate at a density of 20,000 cells per well. After a 7-day osteogenic differential procedure, the alkaline phosphatase (ALP) activity was measured by a ALP activity kit (JianCheng Bioengineering Institute, China) according to the manufacturer’s instructions. The results were normalized to levels of total protein, which were measured by a BCA method (Thermo, USA).

For alkaline phosphatase staining, after 7 days of osteogenic differentiation, the samples were washed three times with PBS solution at room temperature, and the cells were then fixed in 4% paraformaldehyde for 30 min and stained with a 5-bromo-4-chloro-3-indolyl-phosphate (BCIP)/nitro blue tetrazolium (NBT) Alkaline Phosphatase Color Development Kit (Beyotime Institute of Biotechnology China) for 15 min. The cells were washed several times with PBS and analyzed by microscopy.

### Quantitative real-time PCR (q-PCR)

Total mRNA from cells seeded onto a 6-well plate at a density of 80,000 cells per well was isolated by Trizol reagent (Invitrogen, USA). The extracted RNA was quantified with UV spectrophotometry, and only samples with a ratio of absorbance at 260 and 280 nm (the 260/280 ratio) greater than 1.8 were used in the subsequent steps. A PrimeScript RT Reagent Kit (Takara, Japan) was used to reverse-transcribe mRNA (0.5 μg) into cDNA according to the manufacturer’s instructions. FastStart Universal SYBR Green Master Mix (ROX, USA) was mixed with the cDNA, and q-PCR was performed by applying the ABI 7500 Real-Time PCR system (Applied Biosystems, USA). Relative quantization was calculated by the △△Ct method and was normalized to the housekeeping gene glyceraldehyde 3-phosphate dehydrogenase (GAPDH). The sequences of the gene primers used for q-PCR are listed below, including OCN (forward 5′-AGAGCCCCAGTCCCCTACCC-3′ and reverse 5′-AGGCCTCCTGAAAGCCGATG-3′), RUNX2 (forward 5′-CCATAACGGTCTTCACAAATCCT-3′ and reverse 5′-TCTGTCTGTGCCTTCTTGGTTC-3′), OSX (forward 5′-GCGGCAAGGTGTATGGCAAGG-3′ and reverse 5′-GCAGAGCAGGCAGGTGAACTTC-3′), BSP (forward 5′-GTCTATAGAACCACTTCCCCAC-3′ and reverse 5′-GCTGTACTCATCTTCATAGGCT-3′), ALP (forward 5′-CTGGTACTCAGACAACGAGATG-3′ and reverse 5′-GTCAATGTCCCTGATGTTATGC-3′), and GAPDH (forward 5′-GAGTCCACTGGCGTCTTCAC-3′ and reverse 5′-TTCACACCCATGACGAACAT-3′).

### Western blot analysis

The total protein from BMSCs was extracted by lysis in radioimmunoprecipitation assay (RIPA) buffer containing a protease inhibitor cocktail (Solarbio, China). Each group of cell lysates was quantified using a BCA protein kit (Thermo, USA). Approximately 20 mg of protein mixed with loading buffer (Solarbio, China) was separated on 12% Tris-glycine sodium dodecyl sulfate-polyacrylamide gels (SDS-PAGE) in each lane, and the proteins were subsequently transferred onto a polyvinylidene fluoride membrane (Millipore) for immunoblotting. After blocking with 5% skim milk in Tris-buffered saline and Tween 20 (TBST) buffer for 1 h at room temperature, the membrane was incubated with the primary antibodies against rabbit Runx2, β-actin, p-ERK, ERK, p-JNK, JNK, p-P38, and P38 (Abclonal China) overnight at 4 °C. After three washes with TBST, horseradish peroxidase-linked secondary antibodies (Abclonal, China) were used to detect the primary antibodies. The membrane was incubated with the secondary antibodies at room temperature for 1 h. After washing three times with TBST, an enhanced chemiluminescence (ECL) reagent (Abclonal, China) was used to visualize the blots, and ImageJ software (National Institutes of Health, Bethesda, USA) was used to measure the gray value of each target protein.

### Chromatin immunoprecipitation

ChIP assays were explored using a chromatin immunoprecipitation assay kit (Merck Germany) following the manufacturer’s instructions. Approximately 1 × 10^7^ BMSCs for each group were washed with PBS and then fixed on a plate with 1% formaldehyde for 10 min to crosslink DNA–protein complexes. The fixed cells were washed with ice-cold PBS containing protease inhibitors and 1 mM phenylmethylsulfonyl fluoride (PMSF), harvested and resuspended in SDS-lysis buffer containing protease inhibitors and PMSF for 15 min on ice. The isolated nuclei were sonicated using an ultrasonic sonicator (Misonix S-4000, USA) with five 20-s pulses with 45-s intervals to obtain sheared chromatin ranging from 0.2 to 0.6 kb. The supernatants were transferred and diluted with ChIP dilution buffer containing protease inhibitors and PMSF after centrifugation for 15 min at 10000 rpm. For q-PCR analysis, aliquots (1:100) of total chromatin DNA before immunoprecipitation were collected as the input. For immunoprecipitation, the sheared chromatin was incubated with antibodies against Runx2 (Abclonal, China) or immunoglobulin G (IgG) (Millipore, USA) overnight at 4 °C, followed by purification using Protein-A/G Dynabeads. The beads were collected and sequentially washed with the following buffers: low salt wash buffer, high salt wash buffer, LiCl wash buffer, and Tris-EDTA (TE) wash buffer. To elute the DNA, the samples were mixed with elution buffer containing proteinase K at 62 °C for 2 h and 95 °C for 10 min. The supernatants were purified by phenol-chloroform extraction. The precipitated DNA was eluted and amplified using q-PCR. The input lysates were also processed as above. The primers used for real-time PCR were obtained from the OSX, BSP, ALP, and OCN promoter regions.

### Statistical analysis

All data were carried out in triplicate and represented as mean ± standard deviation. *T* tests or one-way analysis of variance (ANOVA) was used, and *P* values < 0.05 were considered statistically significant.

## Results

### D1 and D2 receptors are expressed on BMSCs

**Fig. 1:**
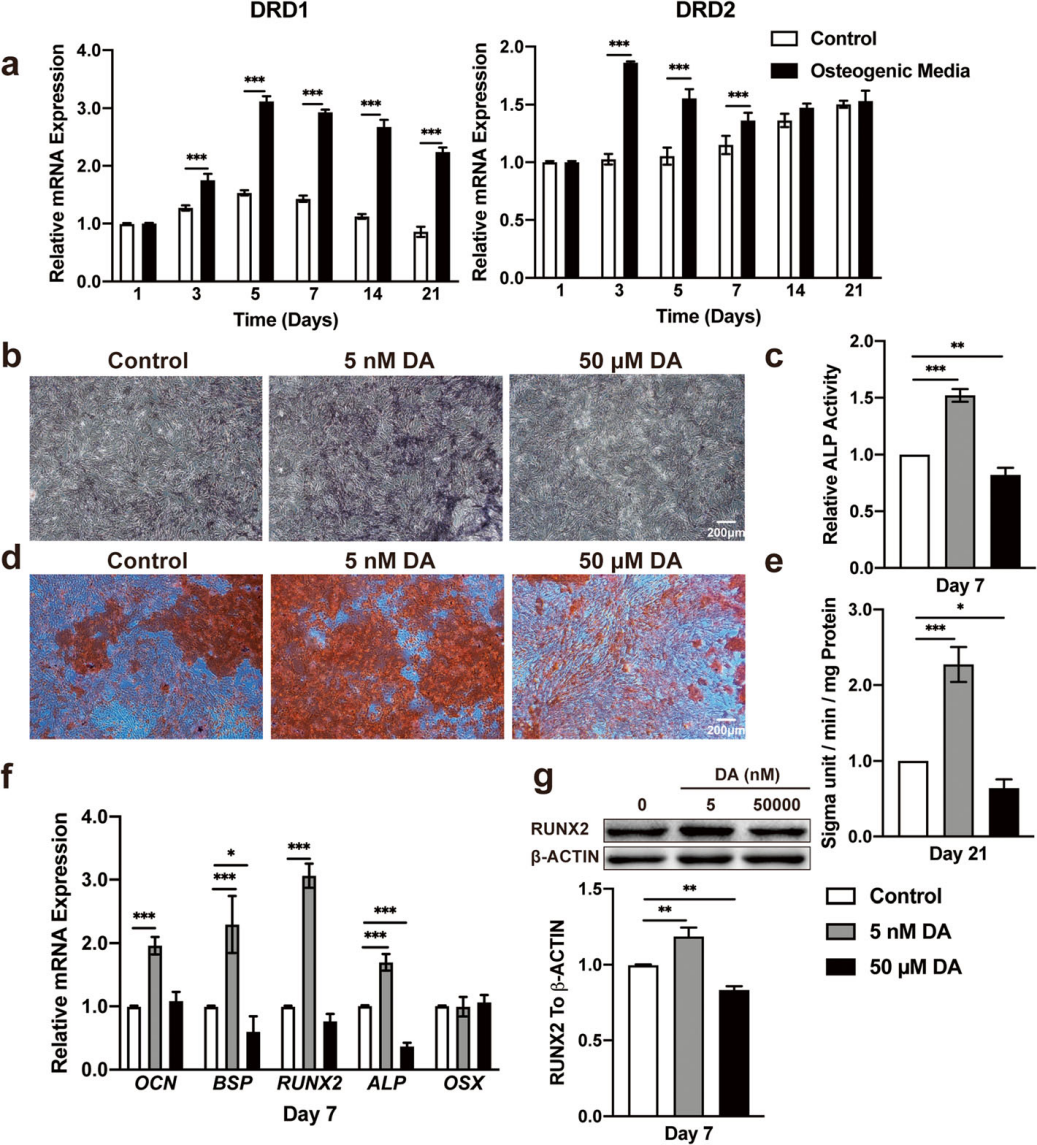
A lower concentration of DA facilitates hBMSC osteogenic differentiation. **a** Quantitative RT-PCR analysis of DRD1 and DRD2 expression during hBMSC osteogenic differentiation on days 1, 3, 5, 7, 14, and 21 (*n* = 3 for all groups). **b** Histochemical staining and **c** total absorbance measurements of ALP during early hBMSC osteogenic differentiation stimulated with DA (*n* = 3 for all groups). **d** Alizarin Red S staining and **e** total absorbance measurements during late hBMSC osteogenic differentiation stimulated with DA (*n* = 3 for all groups). **f** Quantitative RT-PCR analysis of osteogenic gene expression during hBMSC osteogenic differentiation stimulated with DA (*n* = 3 for all groups). **g** Immunoblot analysis of RUNX2 expression during hBMSC osteogenic differentiation stimulated with DA (*n* = 3 for all groups). The results are shown as the mean ± standard error. Statistical significance was assessed by unpaired Student’s *t* test or one-way ANOVA test for multiple-group comparisons; *P < 0.05; **P < 0.01; ***P < 0.001

We investigated the expression of the first two DA receptors, D1 and D2 receptors, on hBMSCs using quantitative RT-PCR. The expression levels of both D1 and D2 receptors remained quite stable from day 1 to day 21 in the culture medium; however, both flattened curves started to increase in osteogenic media for the first 2 days. The D1 receptor increased significantly until day 5, and the D2 receptor decreased from day 3 to day 5. These changes tended to stabilize after day 7, but the cells cultured in osteogenic media maintained higher expression levels than hBMSCs cultured in growth media (Fig. 1a). These results were also performed in rBMSCs from day 1 to day 7 (Additional file 1: Figure S1).

### Different concentrations of DA regulate the differentiation of BMSCs

We performed a concentration–response experiment to determine the safety concentration of DA in hBMSCs and rBMSCs by a Cell Counting Kit-8 (CCK-8) assay. The results demonstrated that the nanomolar range of DA has little influence on hBMSC and rBMSC proliferation, whereas the micromolar range, especially 50 μM, of DA promoted cell proliferation and 500 μM DA significantly inhibited cell proliferation (Additional file 2: Figure S2A and B). The early differentiation of hBMSCs investigated by alkaline phosphatase (ALP) activity assays and ALP staining showed that a safe concentration of 5 nM DA stimulated differentiation, while 50 μM DA markedly inhibited differentiation (Additional file 3: Figure S3A, Fig. 1b, c). The same results were found in rBMSCs (Additional file 3: Figure S3B and C). Final mineralization of hBMSCs, assessed by Alizarin Red S (ARS), showed a similar tendency (Fig. 1d, e). Furthermore, the expression of *BSP*, *ALP*, *Runx2*, and *OCN*, as molecular markers of osteogenesis, was also increased with 5 nM DA and decreased with 50 μM DA by quantitative RT-PCR (Fig. 1f). Western blotting was then used to check Runx2 expression and demonstrated consistent results (Fig. 1g). Therefore, 5 nM DA was used to stimulate osteogenesis of hBMSCs in the following in vitro study.

### Activation of the D1 receptor upregulates the differentiation of hBMSCs

**Fig. 2:**
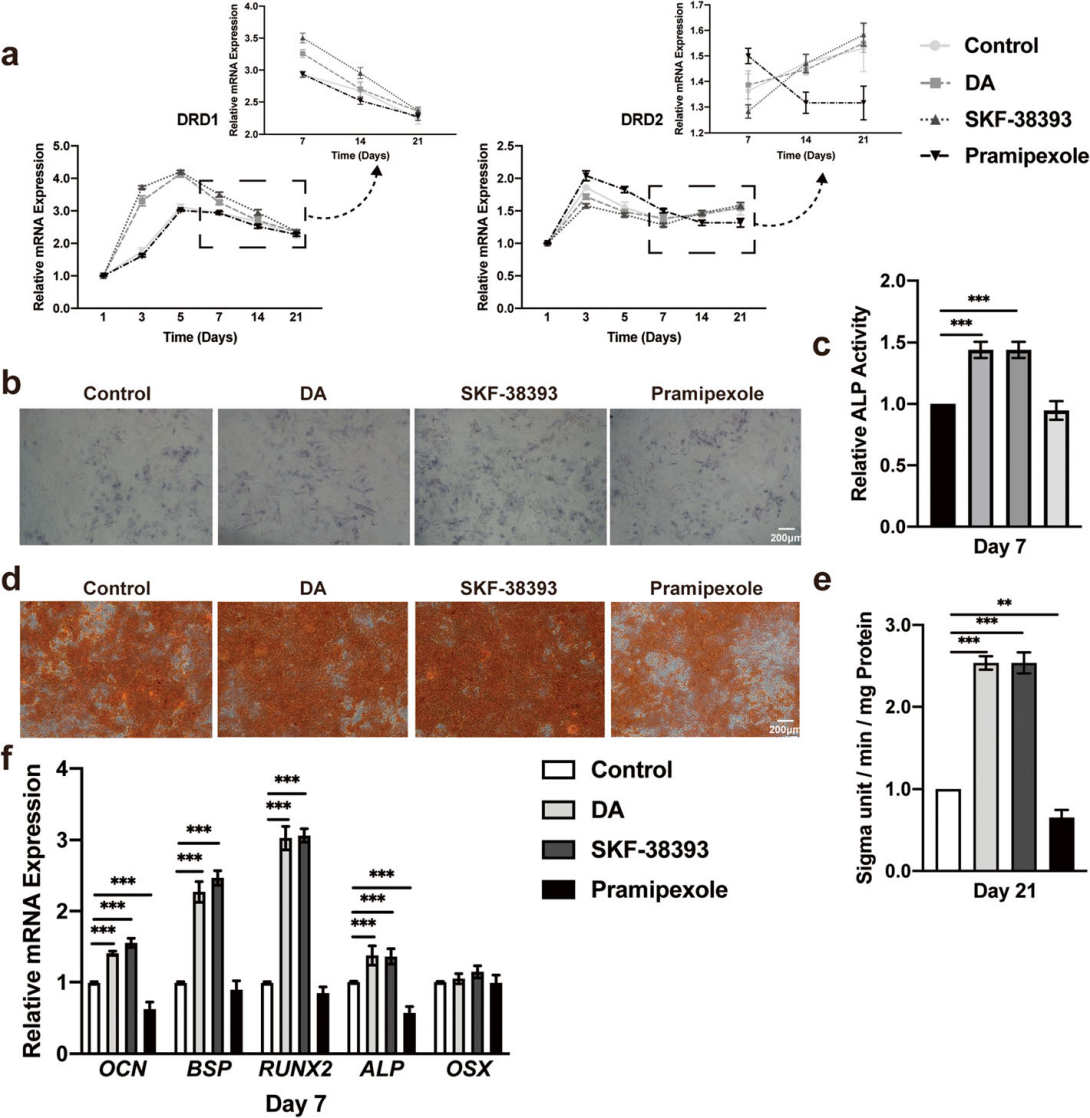
Activation of the D1 receptor promotes hBMSC osteogenic differentiation. **a** Quantitative RT-PCR analysis of DRD1 and DRD2 expression during hBMSC osteogenic differentiation on days 1, 3, 5, 7, 14, and 21 with or without DA, D1 agonist (SKF-38393) and D2 agonist (pramipexole) stimulation (*n* = 3 for all groups). **b** Histochemical staining and **c** total absorbance measurements of ALP during early hBMSC osteogenic differentiation stimulated with DA, SKF-38393, and pramipexole (*n* = 3 for all groups). **d** Alizarin Red S staining and **e** total absorbance measurements during late hBMSC osteogenic differentiation stimulated with DA, SKF-38393, and pramipexole (*n* = 3 for all groups). **f** Quantitative RT-PCR analysis of osteogenic gene expression during hBMSC osteogenic differentiation stimulated with DA, SKF-38393, and pramipexole (*n* = 3 for all groups). The results are shown as the mean ± standard error. Statistical significance was assessed by one-way ANOVA test; *P < 0.05; **P < 0.01; ***P < 0.001

To explore whether DA could activate D1 or D2 receptors to enhance osteogenesis, we first added 5 nM DA, 1 μM SKF-38393, a D1 receptor agonist, or 10 μM pramipexole, a D2 receptor agonist, to the hBMSC culture medium. The concentration of each reagent was carefully chosen to effectively stimulate the differentiation and have little influence on the proliferation of hBMSCs (Additional file 2: Figure S2A, Additional file 3: Figure S3A, Additional file 4: Figure S4 and Additional file 5: Figure S5). Interestingly, adding SKF-38393 or 5 nM DA to osteogenic media further increased D1 receptor expression and decreased D2 receptor expression. However, pramipexole seemed to have the opposite effect (Fig. 2a). The ALP activity of the cells cultured with SKF-38393 was significantly increased, which is comparable to the effect on cells stimulated with DA. Pramipexole seemed to have little influence on hBMSC differentiation (Fig. 2b, c). SKF-38393 and DA also promoted mineralization based on ARS (Fig. 2d, e). Consistent with these results, quantitative real-time PCR analysis demonstrated that the mRNA expression levels of osteogenic markers were only upregulated after SKF-38393 treatment (Fig. 2f).

### Blocking the D1 receptor inhibits hBMSC differentiation and DA effects

**Fig. 3:**
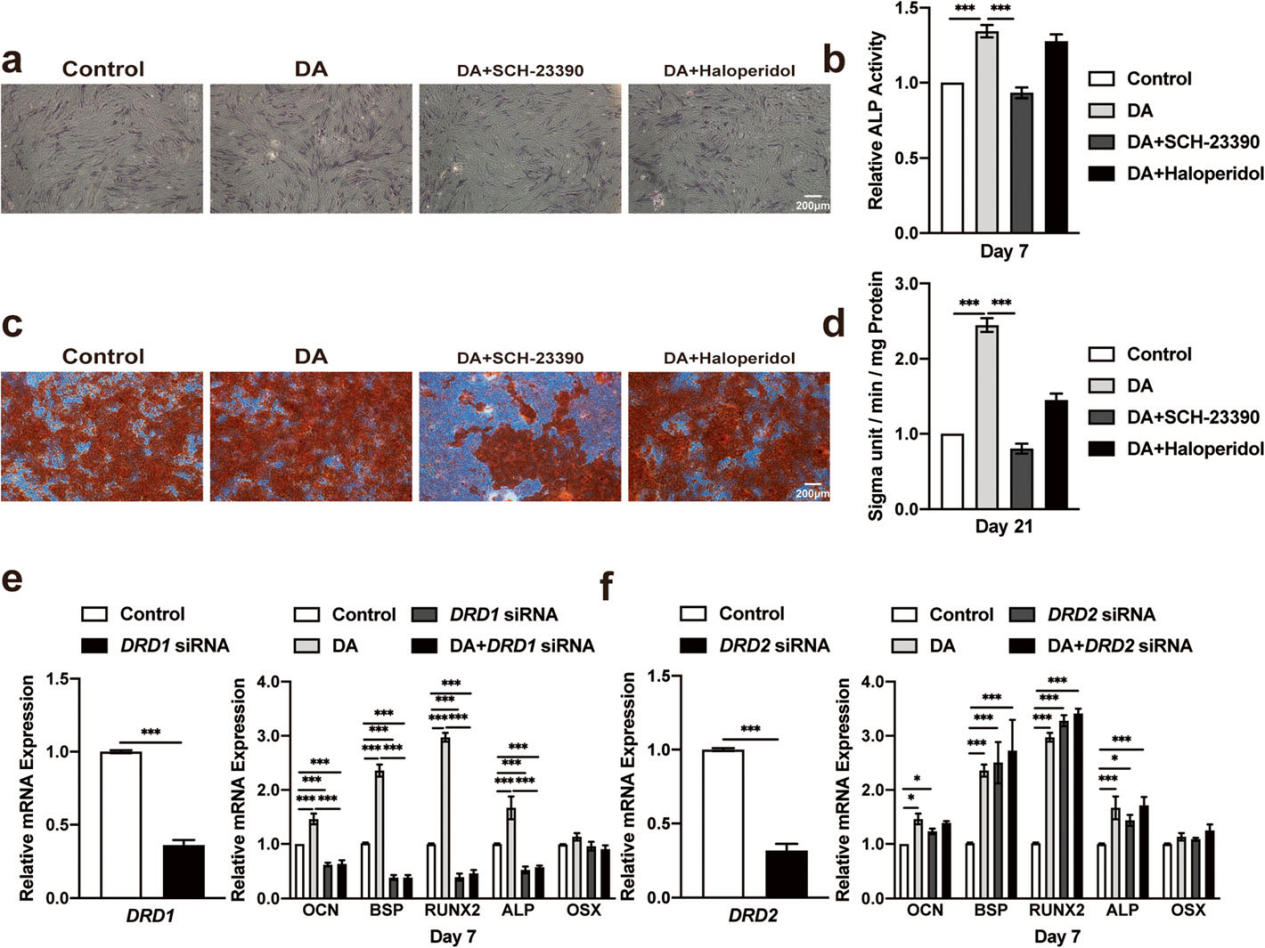
Blocking the D1 receptor inhibits hBMSC osteogenic differentiation. **a** Histochemical staining and **b** total absorbance measurements of ALP during early hBMSC osteogenic differentiation stimulated with DA, DA+D1 antagonist (SCH-23390) and DA+ D2 antagonist (haloperidol) (*n* = 3 for all groups). **c** Alizarin Red S staining and **d** total absorbance measurements during late hBMSC osteogenic differentiation stimulated with DA, DA+SCH-23390 and DA+ haloperidol (*n* = 3 for all groups). Quantitative RT-PCR analysis of osteogenic gene expression during hBMSC osteogenic differentiation after transfection with D1 receptor-specific (**e**) or D2 receptor-specific (**f**) siRNA (*n* = 3 for all groups). The results are shown as the mean ± standard error. Statistical significance was assessed by unpaired Student’s *t* test or one-way ANOVA test for multiple-group comparisons; *P < 0.05; **P < 0.01; ***P < 0.001

To further confirm that the D1 receptor signaling pathway was involved in DA-induced hBMSC osteogenic differentiation, hBMSCs were pretreated with 1 μM SCH-23390 (D1 receptor antagonist) or 20 μM haloperidol (D2 receptor antagonist) for 30 min before stimulation with DA. The concentrations of SCH-23390 and haloperidol were chosen as previously described (Additional file 6: Figure S6 and Additional file 7: Figure S7). After 7 days of culture, DA significantly increased ALP activity. However, this positive effect was impaired by the addition of SCH-23390 but not haloperidol (Fig. 3a, b). SCH-23390 pretreatment also markedly decreased the mineralization of hBMSCs (Fig. 3c, d). We then utilized siRNA transfection to knock down D1 or D2 receptor expression in hBMSCs. The RT-PCR results showed that the DA-induced osteogenesis of hBMSCs was significantly inhibited by D1 receptor knockdown (Fig. 3e, f). Taken together, these results elucidated that the DA-induced osteogenic differentiation of hBMSCs was mediated by the activation of the D1 receptor.

### Blocking the cAMP-PKA signaling pathway inhibits DA-induced differentiation of hBMSCs

**Fig. 4:**
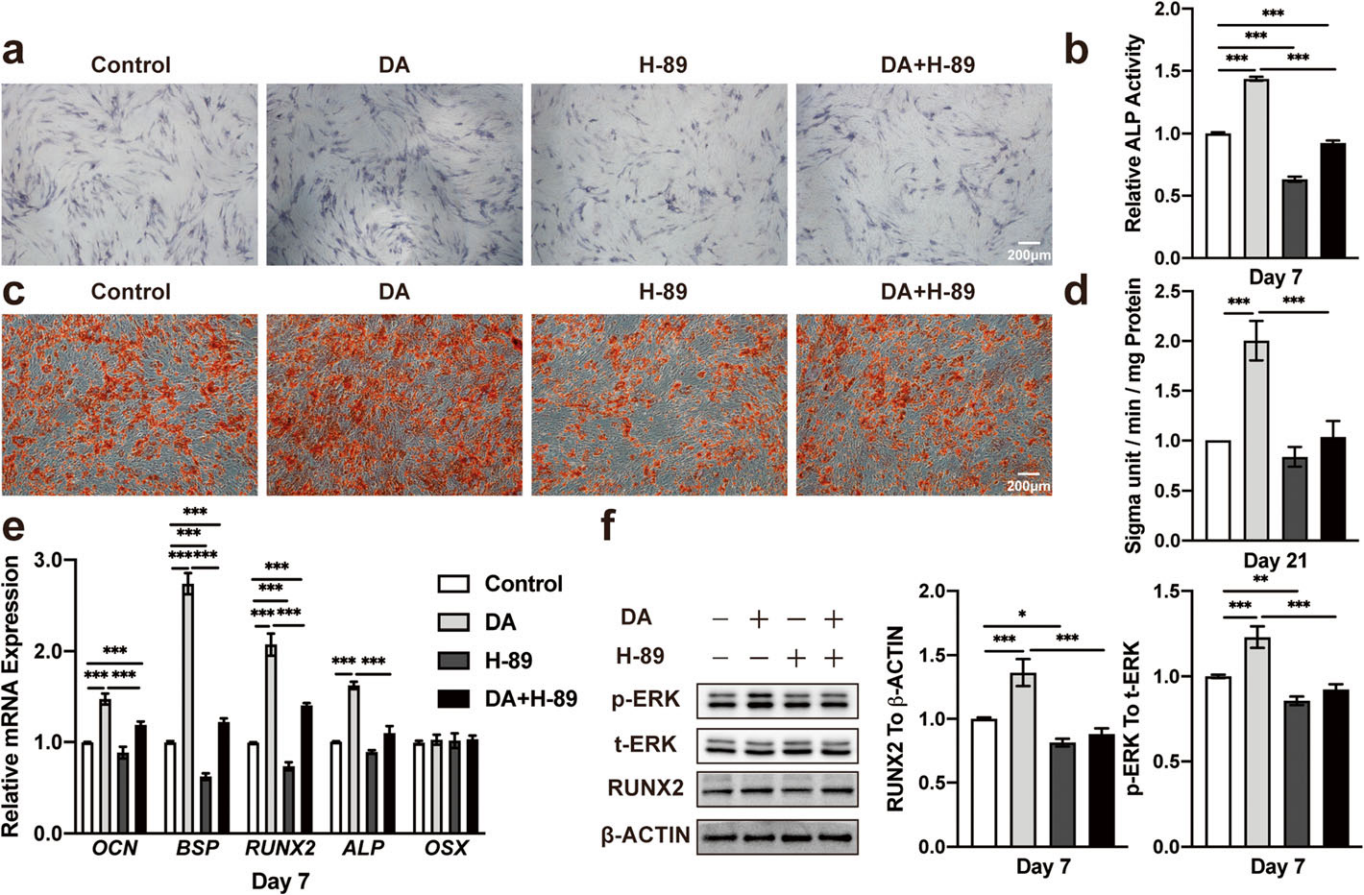
Blocking the cAMP-PKA signaling pathway inhibits ERK1/2 and suppresses hBMSC osteogenic differentiation. **a** Histochemical staining and **b** total absorbance measurements of ALP during early hBMSC osteogenic differentiation stimulated with SKF-38393, PKA inhibitor (H-89), and SKF-38393+ H-89 (*n* = 3 for all groups). **c** Alizarin Red S staining and **d** total absorbance measurements during late hBMSC osteogenic differentiation stimulated with SKF-38393, H-89, and SKF-38393+ H-89 (*n* = 3 for all groups). **e** Quantitative RT-PCR analysis of osteogenic gene expression during hBMSC osteogenic differentiation stimulated with SKF-38393, H-89, and SKF-38393+ H-89 (*n* = 3 for all groups). **f** Immunoblot analysis of Runx2, phosphorylation, and total ERK1/2 expression during hBMSC osteogenic differentiation stimulated with SKF-38393, H-89, and SKF-38393+ H-89 (*n* = 3 for all groups). The results are shown as the mean ± standard error. Statistical significance was assessed by one-way ANOVA test; *P < 0.05; **P < 0.01; ***P < 0.001

Activation of the D1 receptor leads to the phosphorylation of ribosomal protein S6 (rpS6), which is essential for the upregulation of the cAMP-dependent PKA signaling pathway. To determine whether DA stimulation of hBMSC osteogenic differentiation may be mediated through the activation of the cAMP-dependent PKA signaling pathway, we assessed the DA effect after blocking PKA signaling with a selective inhibitor, H-89, at an optimal concentration of 1 μM (Additional file 8: Figure S8), for 30 min before the osteogenesis of hBMSCs. In particular, H-89 at a concentration of 1 μM significantly suppressed DA-induced ALP activity in hBMSCs after 7 days of osteogenesis. However, ALP activity was also decreased with H-89 alone, and there were no significant differences between adding DA after pretreatment with H-89 and using H-89 alone (Fig. 4a, b). The results of ARS staining at day 21 were consistent with ALP activity (Fig. 4c, d). The mRNA expression of osteogenic markers was further enhanced after DA treatment, and the transcriptional promotion by DA was inhibited by H-89 (Fig. 4e). Western blot results showed the same tendency, which suggested that H-89 could suppress the DA-induced osteogenic differentiation of hBMSCs by inhibiting the cAMP-dependent PKA signaling pathway. In addition to the inhibition of Runx2, the Western blot results also showed a remarkable downregulation of ERK1/2 phosphorylation after treatment with H-89 (Fig. 4f). Therefore, the above results suggested that the suppression of the cAMP-dependent PKA signaling pathway may inhibit the DA-induced osteogenic differentiation of hBMSCs.

### Activation of the ERK1/2 signaling pathway seems essential in the DA-induced osteogenic differentiation of hBMSCs via increasing Runx2 transcriptional activity

**Fig. 5:**
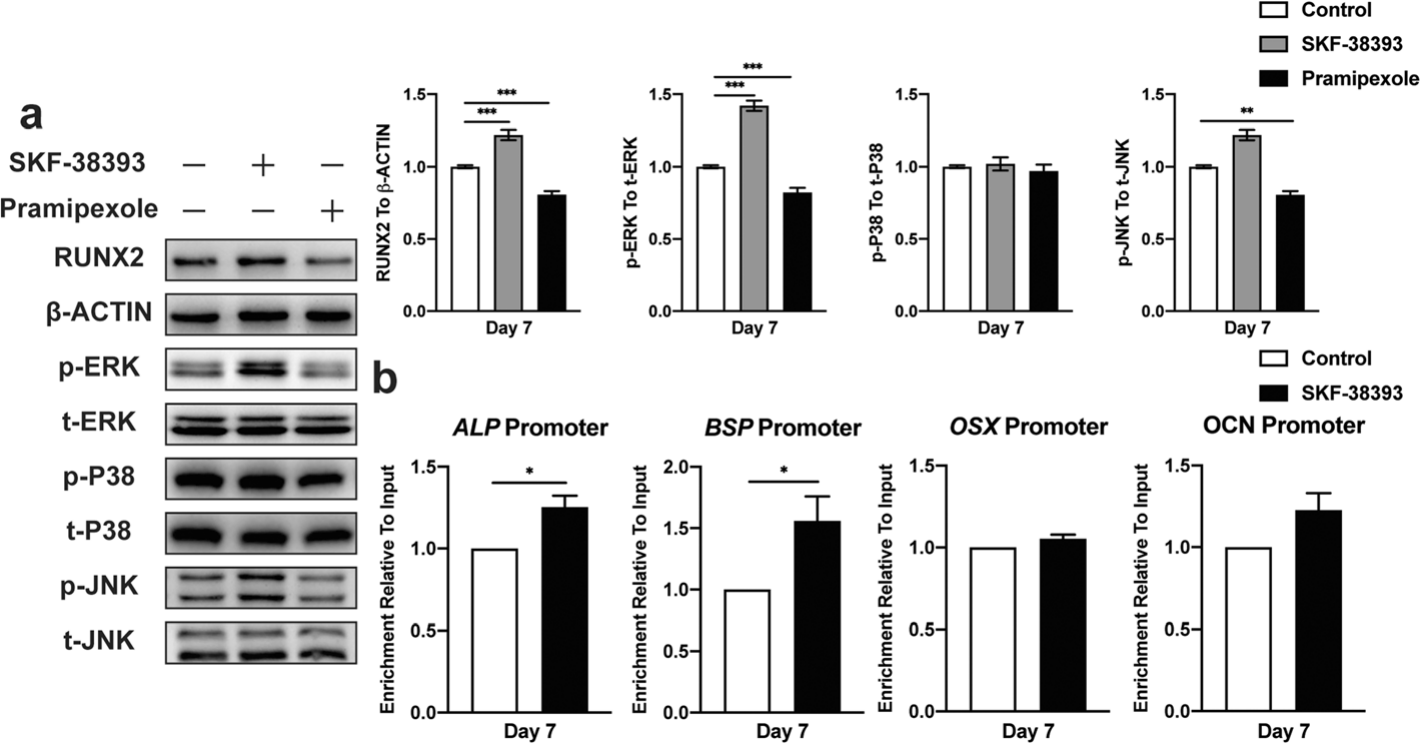
The activation of the D1 receptor enhances ERK1/2 phosphorylation and facilitates hBMSC osteogenic differentiation by increasing Runx2 transcriptional activity. **a** Immunoblot analysis of Runx2, phosphorylation, and total ERK1/2, p38 MAPK, and JNK expression during hBMSC osteogenic differentiation stimulated with SKF-38393 and pramipexole (*n* = 3 for all groups). **b** ChIP assay analysis of Runx2 transcriptional activity in bonding with ALP, BSP, OCN, and OSX promoter during hBMSC osteogenic differentiation stimulated with SKF-38393 (*n* = 3 for all groups). The results are shown as the mean ± standard error. Statistical significance was assessed by unpaired Student’s *t* test or one-way ANOVA test for multiple-group comparisons; *P < 0.05; **P < 0.01; ***P < 0.001

The cAMP-dependent PKA pathway has long been shown to mediate specific intracellular signaling events, including the activation of ERK1/2, JNK, and P38 MAPK, which has been previously reported to lead to osteogenesis. We then investigated whether these events were involved in the DA-induced osteogenic differentiation of hBMSCs. Western blot assays showed that the D1 receptor agonist SKF-38393 could significantly enhance Runx2 expression compared with the expression observed in the control group; however, the D2 receptor agonist pramipexole seemed to have little influence, which was consistent with previous results. There was no remarkable change in JNK and P38 MAPK phosphorylation, and the phosphorylation of ERK1/2 on hBMSCs was significantly increased after SKF-38393 treatment (Fig. 5a). We next assessed the effect of SKF-38393 on Runx2 transcriptional activity and examined whether the upregulation of other osteogenic genes was derived from the stimulation of Runx2. Since Runx2 could physically bind to the promoters of *BSP*, *ALP*, *OCN*, and *OSX*, chromatin immunoprecipitation (ChIP) assays were utilized to analyze the bonding of Runx2 with or without SKF-38393 treatment. After culturing in osteogenic medium for 7 days, adding 1 μM SKF-38393 significantly increased the expression of the promoters of *BSP*, *ALP*, and *OCN* but not *OSX* (Fig. 5b). These results demonstrated that the D1 receptor agonist activated the ERK1/2 signaling pathway and upregulated Runx2 transcriptional activity in hBMSCs, which further mediated the expression of other osteogenic genes.

### Blocking the ERK1/2 signaling pathway inhibited the DA-induced osteogenic differentiation of hBMSCs by suppressing enhanced Runx2 transcriptional activity

**Fig. 6:**
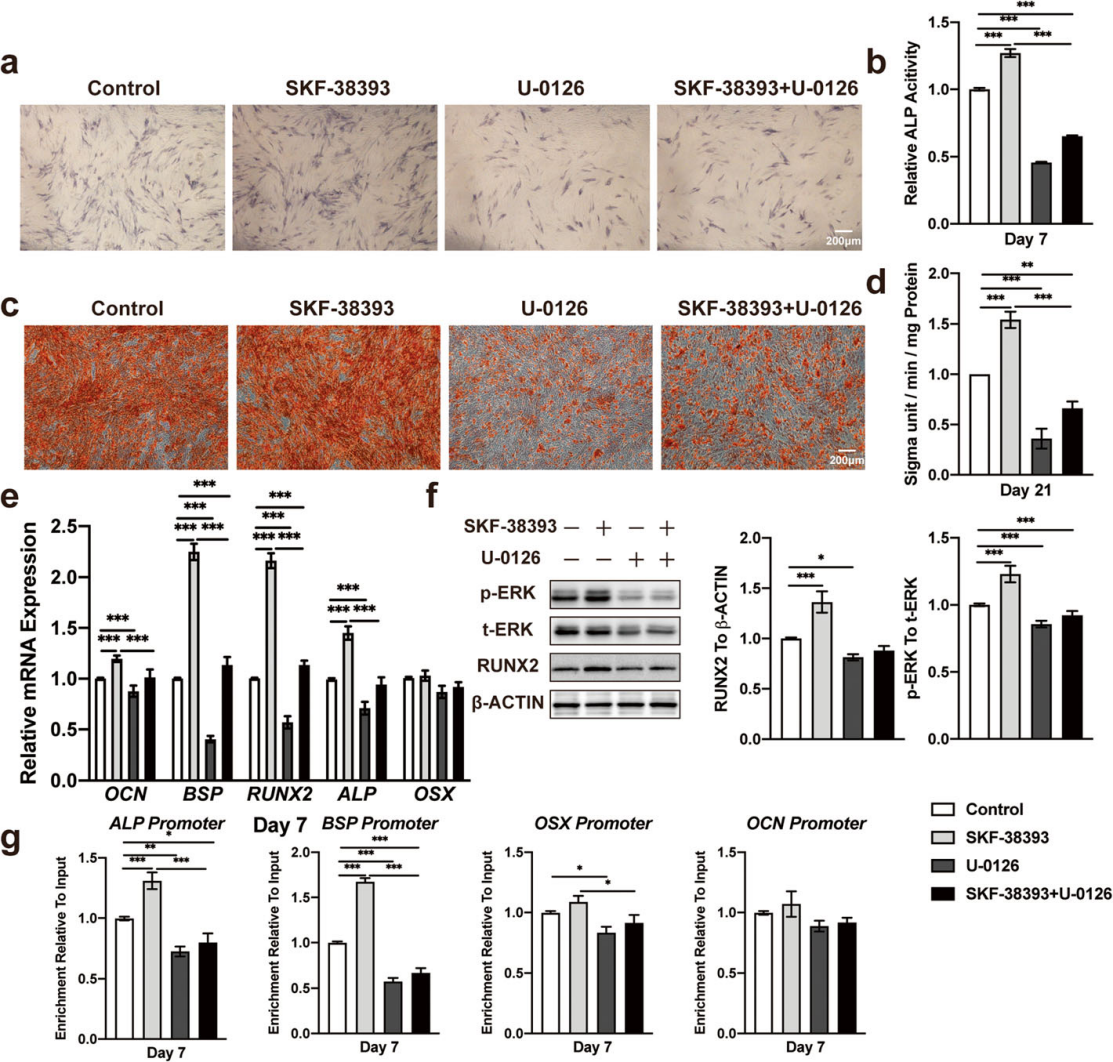
Blocking the ERK1/2 signaling pathway inhibits hBMSC osteogenic differentiation. **a** Histochemical staining and **b** total absorbance measurements of ALP during early hBMSC osteogenic differentiation stimulated with SKF-38393, ERK inhibitor (U-0126), and SKF-38393+ U-0126 (*n* = 3 for all groups). **c** Alizarin Red S staining and **d** total absorbance measurements during late hBMSC osteogenic differentiation stimulated with SKF-38393, U-0126, and SKF-38393+ U-0126 (*n* = 3 for all groups). **e** Quantitative RT-PCR analysis of osteogenic gene expression during hBMSC osteogenic differentiation stimulated with SKF-38393, U-0126, and SKF-38393+ U-0126 (*n* = 3 for all groups). **f** Immunoblot analysis of Runx2, phosphorylation, and total ERK1/2 expression during hBMSC osteogenic differentiation stimulated with SKF-38393, U-0126, and SKF-38393+ U-0126 (*n* = 3 for all groups). **g** ChIP assay analysis of Runx2 transcriptional activity in bonding with ALP, BSP, OCN, and OSX promoter during hBMSC osteogenic differentiation stimulated with SKF-38393, U-0126, and SKF-38393+ U-0126 (*n* = 3 for all groups). The results are shown as the mean ± standard error. Statistical significance was assessed by one-way ANOVA test; *P < 0.05; **P < 0.01; ***P < 0.001

To further verify the relationship between the DA-induced osteogenic differentiation of hBMSCs and ERK1/2 signaling pathway activation and elucidate the role of DA in promoting Runx2 transcriptional activity in hBMSCs, we treated these cells with a selective mitogen-activated protein kinase (MEK)1/2 inhibitor, U-0126, at an optimal concentration of 1 μM (Additional file 9: Figure S9) for 30 min before osteogenic induction. The results showed that ALP activity was significantly suppressed in the group receiving U-0126 alone compared with the untreated control group, and there were no remarkable differences between cells stimulated with SKF-38393 after U-0126 pretreatment and cells treated with U-0126 alone (Fig. 6a, b). The ARS staining results were consistent with ALP activity (Fig. 6c, d). The mRNA expression of osteogenic markers also significantly decreased with U-0126 (Fig. 6e). Western blot results showed that U-0126 successfully suppressed ERK1/2 phosphorylation and inhibited Runx2 expression (Fig. 6f). Moreover, U-0126 also limited DA-induced Runx2 transcriptional activity (Fig. 6g). These results indicated that blocking the ERK1/2 signaling pathway eliminated DA-induced Runx2 transcriptional activity, which led to the inhibition of hBMSC osteogenic differentiation.

## Discussion

In this study, we showed that DA regulated the proliferation and differentiation of BMSCs at different concentrations. Previous research reported that a higher concentration of DA (50 μM) significantly enhanced BMSC adhesion and proliferation, which is consistent with our findings [21]. The effect of DA on osteogenesis via its receptors seemed complicated, and different articles reported contrasting results using different concentrations of DA [13, 20]. This discrepancy might be because DA has a more complex GPCR pharmacology and could in turn mediate several receptors [24]. In addition, studies have recently reported that important differences might exist among individual receptors, providing information to understand the limitations of this and similar cellular models and, moving forward, the cell-specific effects on receptor activity, since trafficking mechanisms may differ substantially among cell types and might be affected by the level of expression of the receptor [31]. Unlike the above studies using MC3T3-E1, a preosteoblast cell line, our results confirmed that a lower concentration of DA (5 nM) could activate the D1 receptor and stimulate the osteogenic differentiation of BMSCs. The slight upregulation of osteogenesis by DA was also found without osteogenic media, which indicated that DA might have an effect on osteogenesis commitment (data not shown). However, the decreased bone mineral density (BMD) and increased fracture risk associated with schizophrenia in patients seem to be counterintuitive based on our results that increased DA upregulated BMSC osteogenic differentiation activity. This paradoxical observation may be due to the notion of DA resistance, which indicated that a DA level above a critical threshold creates a state of resistance to this hormone in BMSCs. Our findings also suggested that DA concentrations above 5 μM inhibit the osteogenic activity of BMSCs, which may occur in a low DA responsiveness situation. The bone is becoming widely recognized as a constantly remodeling endocrine organ; thus, many factors together maintain its homeostasis. DA was reported to be present in the bone marrow, reaching a pharmacology concentration and suppressing osteoclast differentiation [13–15]. Taken together, these findings suggest that the dysregulation of the DA concentration in the bone might tip the balance between osteoblasts and osteoclasts and lead to osteoporosis.

Dysfunctional dopaminergic signaling or its receptors expression level changed could cause several diseases. In the skeletal system, the activation of D2-like receptors could suppress both osteoblast and osteoclast differentiation [14, 32, 33]. Although the exact mechanism for dopaminergic signaling via D1-like receptors remains unclear, previous studies have reported that D1R and D2R signaling are always differentially involved in physiological functions [34], such as regulating the acquisition and retrieval of morphine contextual memory [35]. Furthermore, D1 receptor agonists seem to have comparable effects with D2 receptor antagonists in accelerating bone absorption [33]. This variation was considered to be mainly caused by the opposite cAMP regulation ability of D1 and D2 receptors. Several studies have explored the effects of cAMP on BMSC osteogenic differentiation. Increasing cAMP further led to phosphorylated cAMP response element-binding protein (p-CREB) upregulation, which promoted osteogenesis, whereas inhibiting cAMP could also activate the BMP signaling pathway and thus have the same function [31, 36]. These findings indicated that cAMP influences bone formation through multiple pathways. Our current results suggested that the optimal concentration of DA leads to the activation of the D1 receptor-induced osteogenic differentiation of BMSCs by upregulating cAMP.

A study confirmed that MAPK/ERK acted downstream of GPCRs by reporting that G protein (Rgs12) knockdown induced downregulate of cAMP level only been rescued by overexpressing Rgs12 but not introducing MAPK/ERK activation (MEK1DD transfection) [37]. Roof et al. reported that dopamine receptor activation results in ERK stimulation and contributes to maintain lactotrope homeostasis [38]. Besides, previous studies have reported that MAPK/ERK induced the activation of Runx2, suggesting that the MAPK/ERK signaling pathway might have a positive effect on osteogenesis [39–41]. Therefore, we elucidated the MAPK/ERK signaling pathway underlying the effects of DA on BMSC osteogenic differentiation. Interestingly, the results showed that the D1 receptor agonist selectively activated ERK rather than the JNK and P38 pathways in BMSCs, and different cell types might have influences on this activity [42].

This current study has some limitations. First, both the D1 receptor and D5 receptor increase the concentration of cAMP and are activated by DA. Recently, there has been mounting evidence indicating that several GPCRs can exist in oligomeric forms, making the traditional binding between ligand and receptor much more complicated [43]. Although we did not measure the direct affinity between DA and its receptors, their pharmacological binding sites are not exactly the same, and their affinities also vary significantly. The current evidence based on our results should be sufficient to verify the dominant role of the D1 receptor on BMSC osteogenic differentiation. Second, DA receptors changed under the stimulation of DA or specific agonists, highlighting the complexity of the metabolic consequences of DA receptors. Some articles have previously reported that DA receptors could be internalized intracellularly. Other studies have reported that GPCRs, such as thyrotropin (TSH) receptor, could be internalized intracellularly to regulate osteogenesis via G_s_-protein signaling second-step activation and thus lead to cAMP stimulation [44]. Further research is needed to demonstrate this process.

In conclusion, the present study showed, for the first time, that an appropriate concentration of DA could activate the D1 receptor on BMSCs and further promote osteogenesis via the activation of ERK signaling pathway. Understanding the direct regulation of DA on BMSCs and the underlying mechanisms provides a better awareness of the relationship between neuropsychiatric disorders and osteoporosis and might suggest a novel therapeutic strategy for bone regeneration.

## Supplementary information

**Additional file 1 : Figure S1.** The mRNA expression level of DA receptors, DRD1 and DRD2, increased during osteogenic differentiation of rBMSCs. Quantitative RT-PCR analysis of DRD1 (A) and DRD2 (B) expression during rBMSCs osteogenic differentiation on days 1, 3, 5, 7 (*n* = 3 for all groups). Statistical significance was assessed by unpaired Student’s t test; *P < 0.05; **P < 0.01; ***P < 0.001.

**Additional file 2 : Figure S2.** Different concentration of DA on hBMSCs and rBMSCs proliferation using CCK-8. (A) CCK-8 analysis of hBMSCs treated with various concentrations (0, 0.5, 5, 50, 500 nmol/L and 5, 50, 500 μmol/L) of DA on days 1, 3, 5, and 7 (*n* = 3 for all groups). (B) CCK-8 analysis of rBMSCs treated with various concentrations (0, 0.5, 5, 50, 500 nmol/L and 5, 50, 500 μmol/L) of DA on days 1, 3, 5, and 7 (n = 3 for all groups). Statistical significance was assessed by One-way ANOVA test; *P < 0.05; **P < 0.01; ***P < 0.001.

**Additional file 3 : Figure S3.** Optimization of the concentration of DA on hBMSCs and rBMSCs differentiation using ALP activity assay and ALP staining. (A) ALP activity assay evaluation of hBMSCs osteogenic differentiation under the concentration (0, 0.5, 5, 50, 500 nmol/L and 5, 50, 500 μmol/L) of DA on day 7 (*n* = 3 for all groups). (B) ALP activity assay evaluation of rBMSCs osteogenic differentiation under the concentration (0, 0.5, 5, 50, 500 nmol/L and 5, 50, 500 μmol/L) of DA on day 7 (n = 3 for all groups). (C) Histochemical staining of ALP during early rBMSC osteogenic differentiation stimulated with DA (*n* = 3 for all groups) Statistical significance was assessed by One-way ANOVA test; *P < 0.05; **P < 0.01; ***P < 0.001.

**Additional file 4 : Figure S4.** Optimization of the concentration of SKF-38393, a D1 receptor agonist, on hBMSCs using CCK-8 and ALP activity assays. (A) CCK-8 analysis of hBMSCs treated with various concentrations (0, 1, 10, 100 nmol/L and 1, 10, 20, 50 μmol/L) of DA on days 1, 3, 5, and 7 (*n* = 3 for all groups). (B) ALP activity assay evaluation of hBMSCs osteogenic differentiation under the same concentration of DA on day 7 (*n* = 3 for all groups). Statistical significance was assessed by One-way ANOVA test; *P < 0.05; **P < 0.01; ***P < 0.001.

**Additional file 5 : Figure S5.** Optimization of the concentration of pramipexole, a D2 receptor agonist, on hBMSCs using CCK-8 and ALP activity assays. (A) CCK-8 analysis of hBMSCs treated with various concentrations (0, 1, 10, 100 nmol/L and 1, 10, 20, 50 μmol/L) of DA on days 1, 3, 5, and 7 (*n* = 3 for all groups). (B) ALP activity assay evaluation of hBMSCs osteogenic differentiation under the same concentration of DA on day 7 (*n* = 3 for all groups). Statistical significance was assessed by One-way ANOVA test; *P < 0.05; **P < 0.01; ***P < 0.001.

**Additional file 6 : Figure S6.** Optimization of the concentration of SCH-23390, a D1 receptor antagonist, on hBMSCs using CCK-8 and ALP activity assays. (A) CCK-8 analysis of hBMSCs treated with various concentrations (0, 1, 10, 100 nmol/L and 1, 10, 20, 50 μmol/L) of DA on days 1, 3, 5, and 7 (*n* = 3 for all groups). (B) ALP activity assay evaluation of hBMSCs osteogenic differentiation under the same concentration of DA on day 7 (*n* = 3 for all groups). Statistical significance was assessed by One-way ANOVA test; *P < 0.05; **P < 0.01; ***P < 0.001.

**Additional file 7 : Figure S7.** Optimization of the concentration of haloperidol, a D2 receptor antagonist, on hBMSCs using CCK-8 and ALP activity assays. (A) CCK-8 analysis of hBMSCs treated with various concentrations (0, 1, 10, 100 nmol/L and 1, 10, 20, 50 μmol/L) of DA on days 1, 3, 5, and 7 (*n* = 3 for all groups). (B) ALP activity assay evaluation of hBMSCs osteogenic differentiation under the same concentration of DA on day 7 (n = 3 for all groups). Statistical significance was assessed by One-way ANOVA test; *P < 0.05; **P < 0.01; ***P < 0.001.

**Additional file 8 : Figure S8.** Optimization of the concentration of H-89, a PKA inhibitor, on hBMSCs using CCK-8 and ALP activity assays. (A) CCK-8 analysis of hBMSCs treated with various concentrations (0, 1, 10, 100 nmol/L and 1, 5, 10, 20 μmol/L) of DA on days 1, 3, 5, and 7 (n = 3 for all groups). (B) ALP activity assay evaluation of hBMSCs osteogenic differentiation under the same concentration of DA on day 7 (n = 3 for all groups). Statistical significance was assessed by One-way ANOVA test; *P < 0.05; **P < 0.01; ***P < 0.001.

**Additional file 9 : Figure S9.** Optimization of the concentration of U0126, a MEK1/2 inhibitor, on hBMSCs using CCK-8 and ALP activity assays. (A) CCK-8 analysis of hBMSCs treated with various concentrations (0, 1, 10, 100 nmol/L and 1, 5, 10, 20 μmol/L) of DA on days 1, 3, 5, and 7 (n = 3 for all groups). (B) ALP activity assay evaluation of hBMSCs osteogenic differentiation under the same concentration of DA on day 7 (n = 3 for all groups). Statistical significance was assessed by One-way ANOVA test; *P < 0.05; **P < 0.01; ***P < 0.001.

## Data Availability

All data generated or analyzed during this study are included in this published article [and its supplementary information files].

## Abbreviations

BMSCs: Bone mesenchymal stem cells
hBMSCs: Human bone mesenchymal stem cells
rBMSCs: Rat bone mesenchymal stem cells
DA: Dopamine
PKA: Protein kinase A
SNS: Sympathetic nervous system
β2AR: β2-adrenergic receptor
GPCRs: G protein-coupled receptors
DAR: Dopamine receptor
PTHR: Parathyroid hormone receptor
CaSR: Calcium-sensing receptor
5-HTR: 5-Hydroxytryptamine receptor
CNS: Central nervous system
DAT: Dopamine transporter
RIS: Risperidone
OVX: Ovariectomized
cAMP: Cyclic adenosine monophosphate
MAPKs: Mitogen-activated protein kinases
ERK1/2: Extracellular signal-regulated kinase 1/2
JNK1-3: c-Jun N-terminal kinases 1-3
p38α: β, γ, and δ, p38 isoforms
α-MEM: α-Minimal Essential Media
FBS: Fetal bovine serum
BCA: Bicinchoninic acid
ALP: Alkaline phosphatase
q-PCR: Quantitative real-time PCR
GAPDH: Glyceraldehyde 3-phosphate dehydrogenase
ARS: Alizarin Red S
ChIP: Chromatin immunoprecipitation
