# Supplementary figures and images for: Dopamine D1 receptor-mediated activation of the ERK signaling pathway is involved in the osteogenic differentiation of bone mesenchymal stem cells
Source: Stem Cell Res Ther. 2020 Jan 3;11:12. doi: 10.1186/s13287-019-1529-x (PMC6942280; doi:10.1186/s13287-019-1529-x)

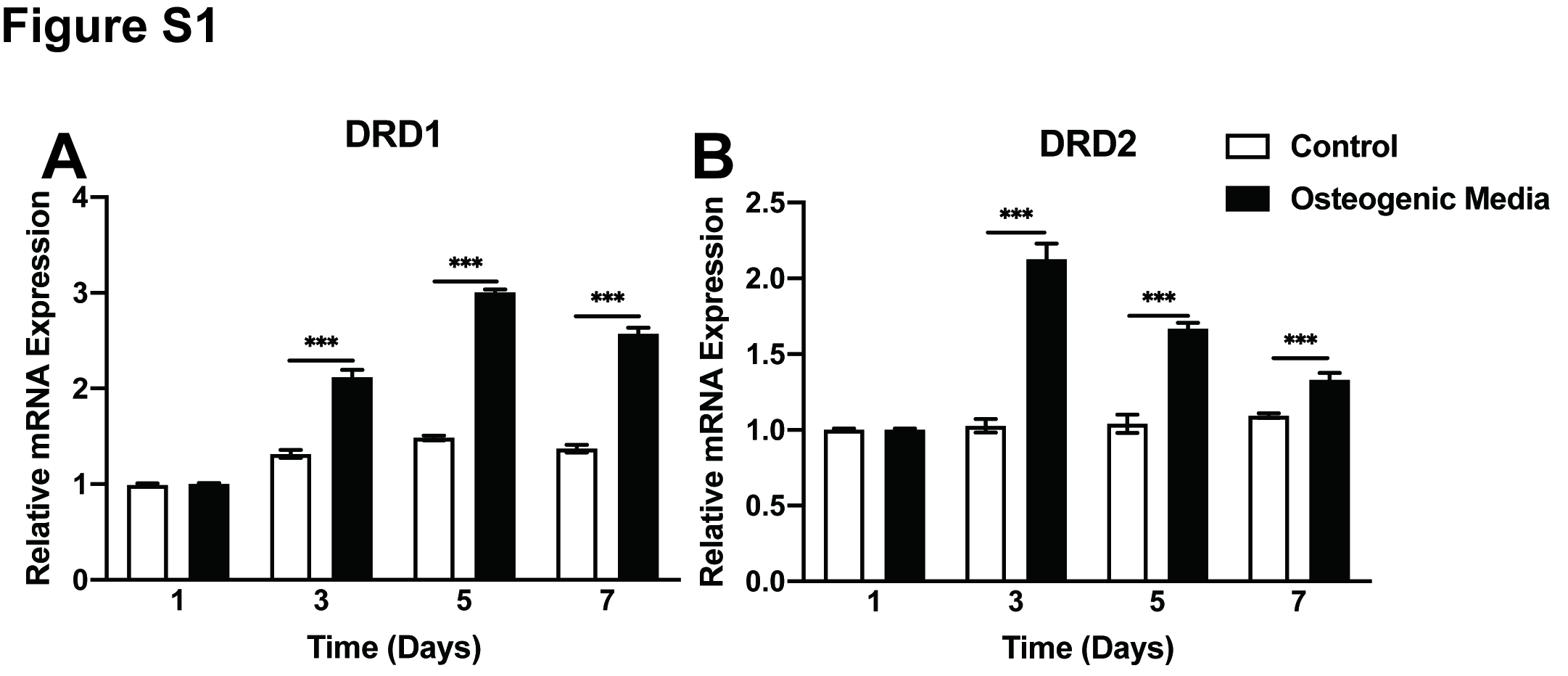

Supplement: Supplementary file 1 — Additional file 1 : Figure S1. The mRNA expression level of DA receptors, DRD1 and DRD2, increased during osteogenic differentiation of rBMSCs. Quantitative RT-PCR analysis of DRD1 (A) and DRD2 (B) expression during rBMSCs osteogenic differentiation on days 1, 3, 5, 7 (n = 3 for all groups). Statistical significance was assessed by unpaired Student’s t test; *P < 0.05; **P < 0.01; ***P < 0.001. [file 13287_2019_1529_MOESM1_ESM.tif]

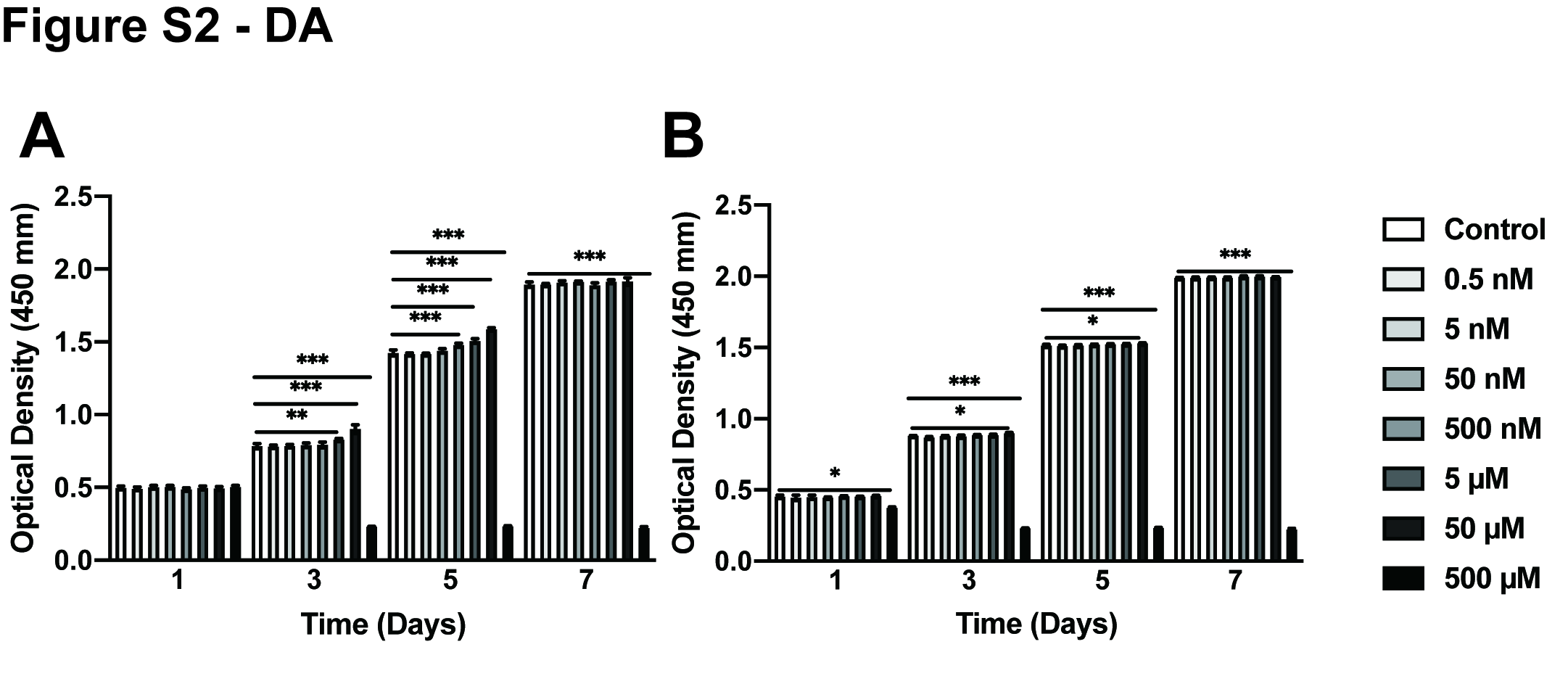

Supplement: Supplementary file 2 — Additional file 2 : Figure S2. Different concentration of DA on hBMSCs and rBMSCs proliferation using CCK-8. (A) CCK-8 analysis of hBMSCs treated with various concentrations (0, 0.5, 5, 50, 500 nmol/L and 5, 50, 500 μmol/L) of DA on days 1, 3, 5, and 7 (n = 3 for all groups). (B) CCK-8 analysis of rBMSCs treated with various concentrations (0, 0.5, 5, 50, 500 nmol/L and 5, 50, 500 μmol/L) of DA on days 1, 3, 5, and 7 (n = 3 for all groups). Statistical significance was assessed by One-way ANOVA test; *P < 0.05; **P < 0.01; ***P < 0.001. [file 13287_2019_1529_MOESM2_ESM.tif]

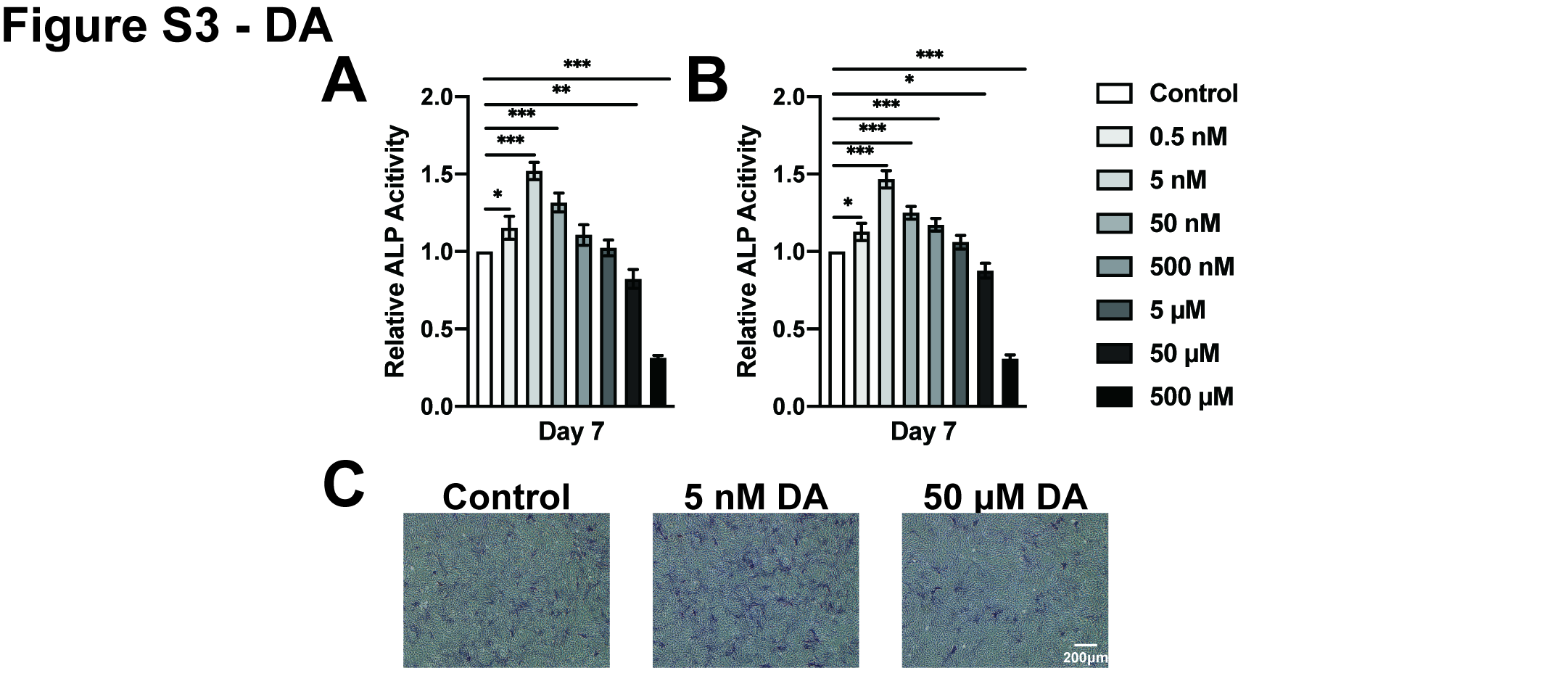

Supplement: Supplementary file 3 — Additional file 3 : Figure S3. Optimization of the concentration of DA on hBMSCs and rBMSCs differentiation using ALP activity assay and ALP staining. (A) ALP activity assay evaluation of hBMSCs osteogenic differentiation under the concentration (0, 0.5, 5, 50, 500 nmol/L and 5, 50, 500 μmol/L) of DA on day 7 (n = 3 for all groups). (B) ALP activity assay evaluation of rBMSCs osteogenic differentiation under the concentration (0, 0.5, 5, 50, 500 nmol/L and 5, 50, 500 μmol/L) of DA on day 7 (n = 3 for all groups). (C) Histochemical staining of ALP during early rBMSC osteogenic differentiation stimulated with DA (n = 3 for all groups) Statistical significance was assessed by One-way ANOVA test; *P < 0.05; **P < 0.01; ***P < 0.001. [file 13287_2019_1529_MOESM3_ESM.tif]

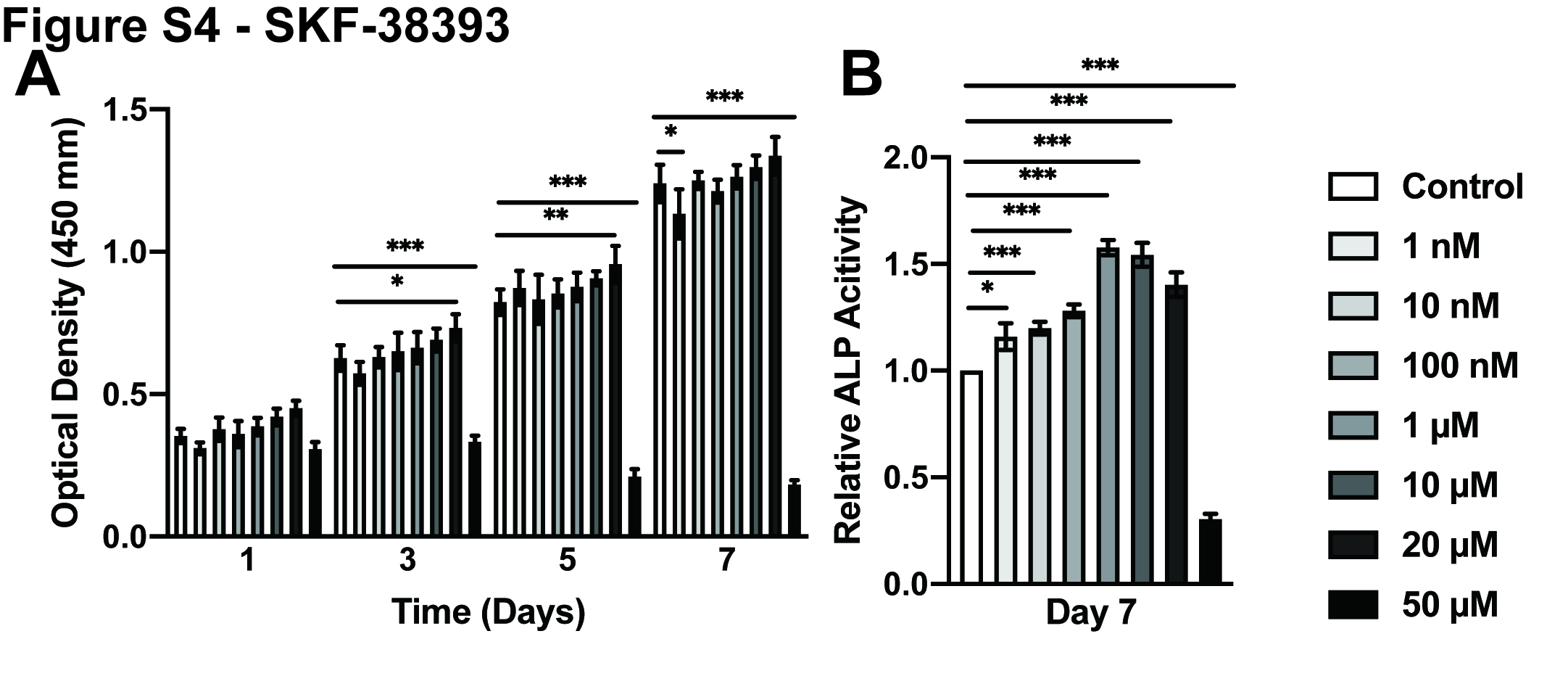

Supplement: Supplementary file 4 — Additional file 4 : Figure S4. Optimization of the concentration of SKF-38393, a D1 receptor agonist, on hBMSCs using CCK-8 and ALP activity assays. (A) CCK-8 analysis of hBMSCs treated with various concentrations (0, 1, 10, 100 nmol/L and 1, 10, 20, 50 μmol/L) of DA on days 1, 3, 5, and 7 (n = 3 for all groups). (B) ALP activity assay evaluation of hBMSCs osteogenic differentiation under the same concentration of DA on day 7 (n = 3 for all groups). Statistical significance was assessed by One-way ANOVA test; *P < 0.05; **P < 0.01; ***P < 0.001. [file 13287_2019_1529_MOESM4_ESM.tif]

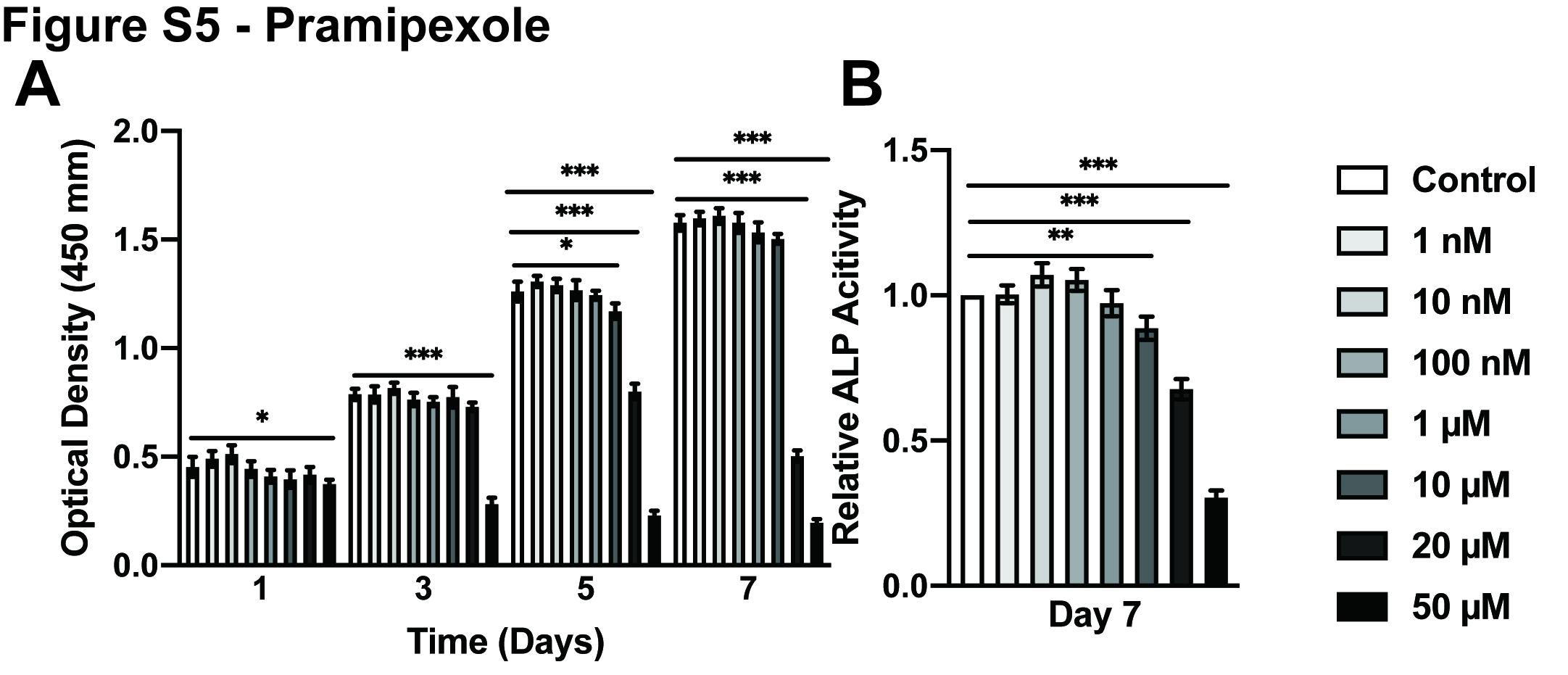

Supplement: Supplementary file 5 — Additional file 5 : Figure S5. Optimization of the concentration of pramipexole, a D2 receptor agonist, on hBMSCs using CCK-8 and ALP activity assays. (A) CCK-8 analysis of hBMSCs treated with various concentrations (0, 1, 10, 100 nmol/L and 1, 10, 20, 50 μmol/L) of DA on days 1, 3, 5, and 7 (n = 3 for all groups). (B) ALP activity assay evaluation of hBMSCs osteogenic differentiation under the same concentration of DA on day 7 (n = 3 for all groups). Statistical significance was assessed by One-way ANOVA test; *P < 0.05; **P < 0.01; ***P < 0.001. [file 13287_2019_1529_MOESM5_ESM.tif]

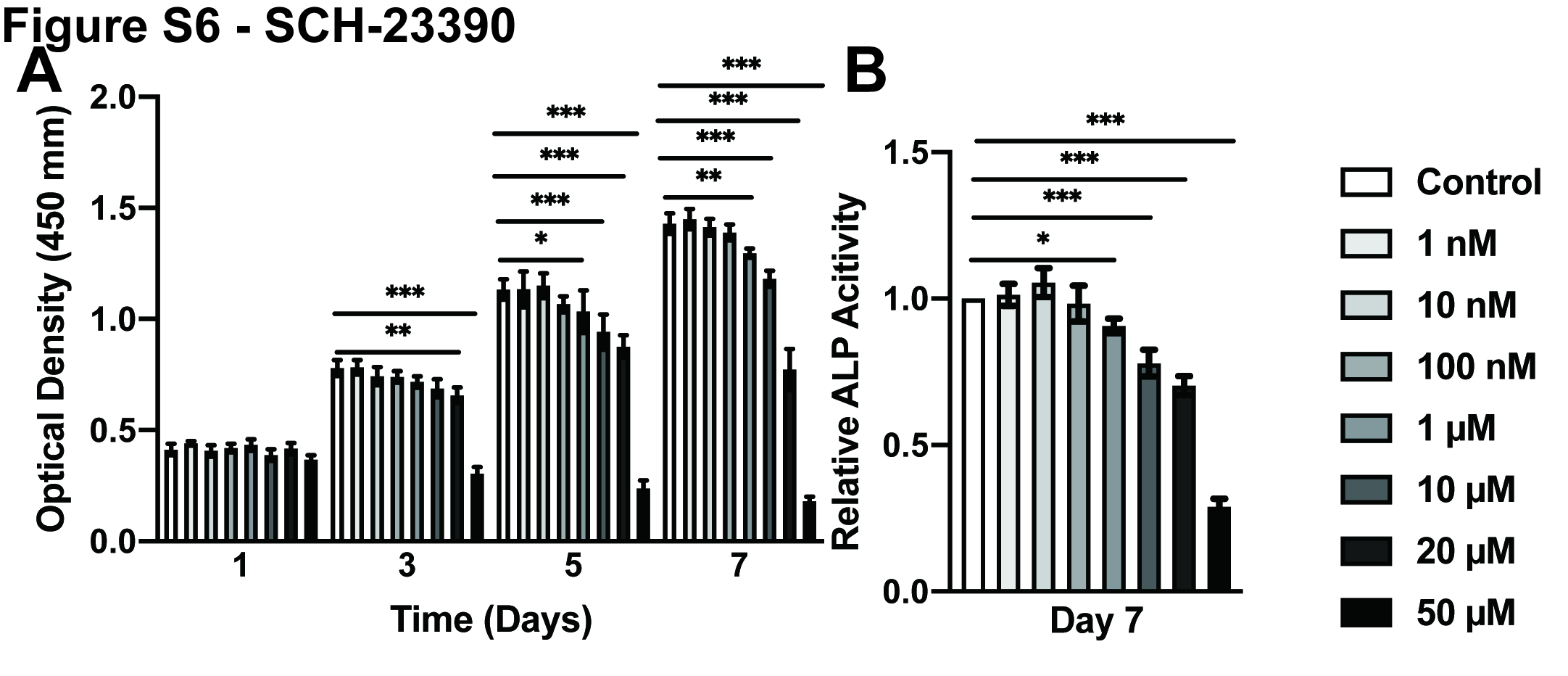

Supplement: Supplementary file 6 — Additional file 6 : Figure S6. Optimization of the concentration of SCH-23390, a D1 receptor antagonist, on hBMSCs using CCK-8 and ALP activity assays. (A) CCK-8 analysis of hBMSCs treated with various concentrations (0, 1, 10, 100 nmol/L and 1, 10, 20, 50 μmol/L) of DA on days 1, 3, 5, and 7 (n = 3 for all groups). (B) ALP activity assay evaluation of hBMSCs osteogenic differentiation under the same concentration of DA on day 7 (n = 3 for all groups). Statistical significance was assessed by One-way ANOVA test; *P < 0.05; **P < 0.01; ***P < 0.001. [file 13287_2019_1529_MOESM6_ESM.tif]

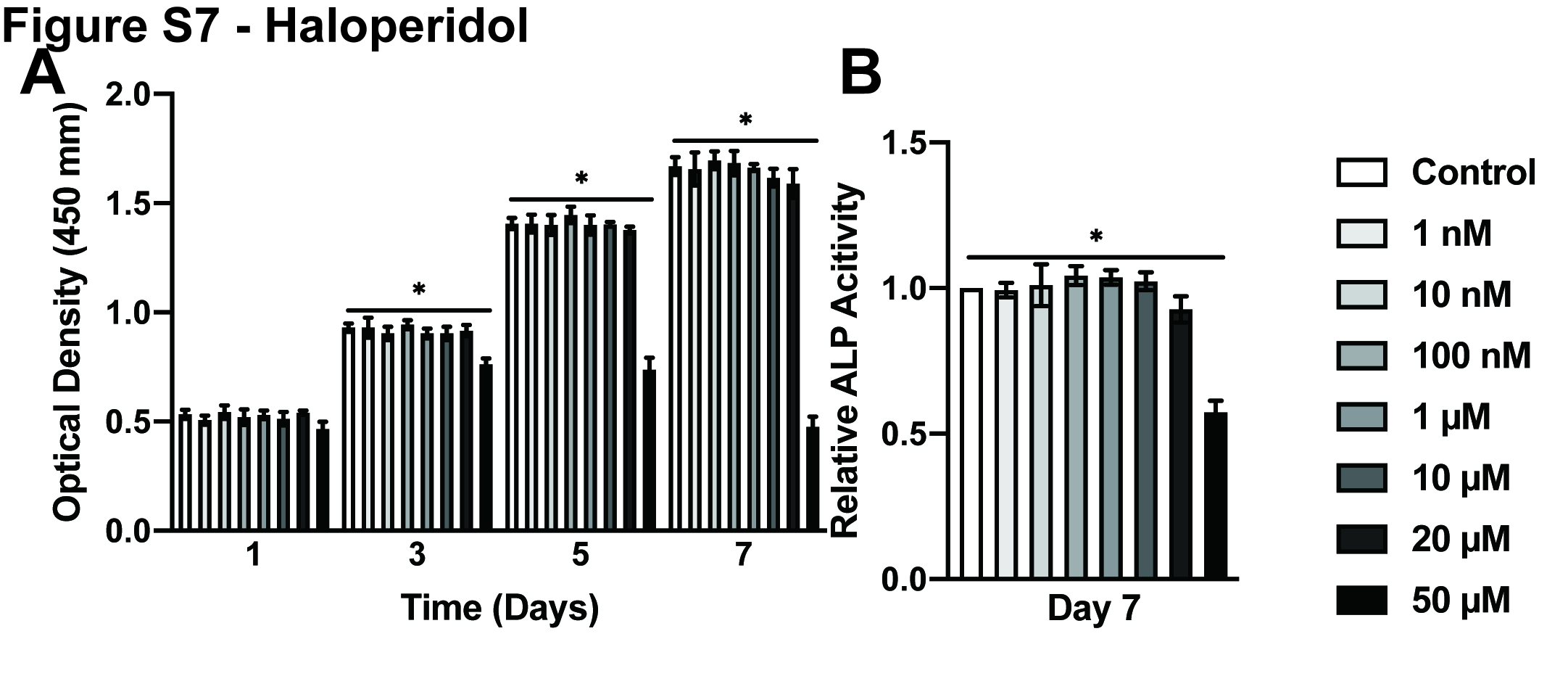

Supplement: Supplementary file 7 — Additional file 7 : Figure S7. Optimization of the concentration of haloperidol, a D2 receptor antagonist, on hBMSCs using CCK-8 and ALP activity assays. (A) CCK-8 analysis of hBMSCs treated with various concentrations (0, 1, 10, 100 nmol/L and 1, 10, 20, 50 μmol/L) of DA on days 1, 3, 5, and 7 (n = 3 for all groups). (B) ALP activity assay evaluation of hBMSCs osteogenic differentiation under the same concentration of DA on day 7 (n = 3 for all groups). Statistical significance was assessed by One-way ANOVA test; *P < 0.05; **P < 0.01; ***P < 0.001. [file 13287_2019_1529_MOESM7_ESM.tif]

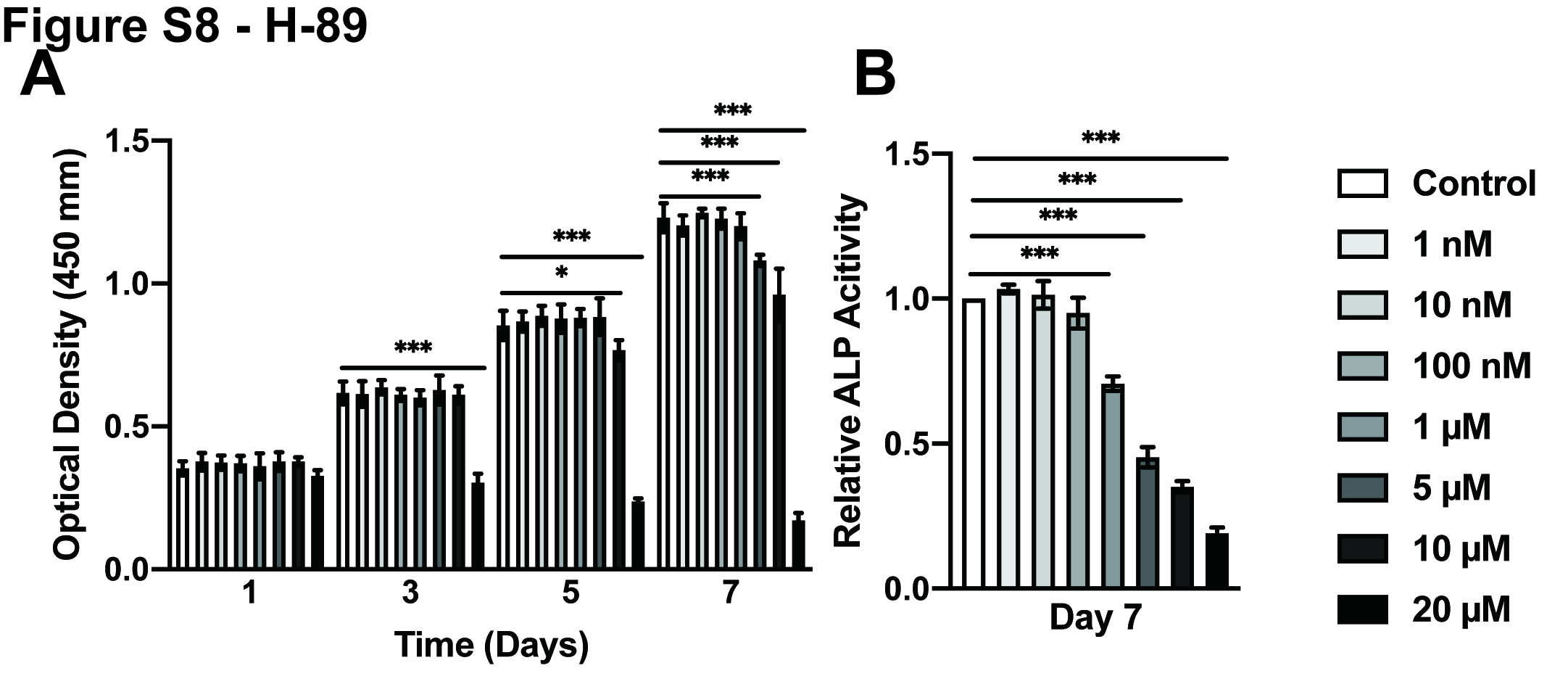

Supplement: Supplementary file 8 — Additional file 8 : Figure S8. Optimization of the concentration of H-89, a PKA inhibitor, on hBMSCs using CCK-8 and ALP activity assays. (A) CCK-8 analysis of hBMSCs treated with various concentrations (0, 1, 10, 100 nmol/L and 1, 5, 10, 20 μmol/L) of DA on days 1, 3, 5, and 7 (n = 3 for all groups). (B) ALP activity assay evaluation of hBMSCs osteogenic differentiation under the same concentration of DA on day 7 (n = 3 for all groups). Statistical significance was assessed by One-way ANOVA test; *P < 0.05; **P < 0.01; ***P < 0.001. [file 13287_2019_1529_MOESM8_ESM.tif]

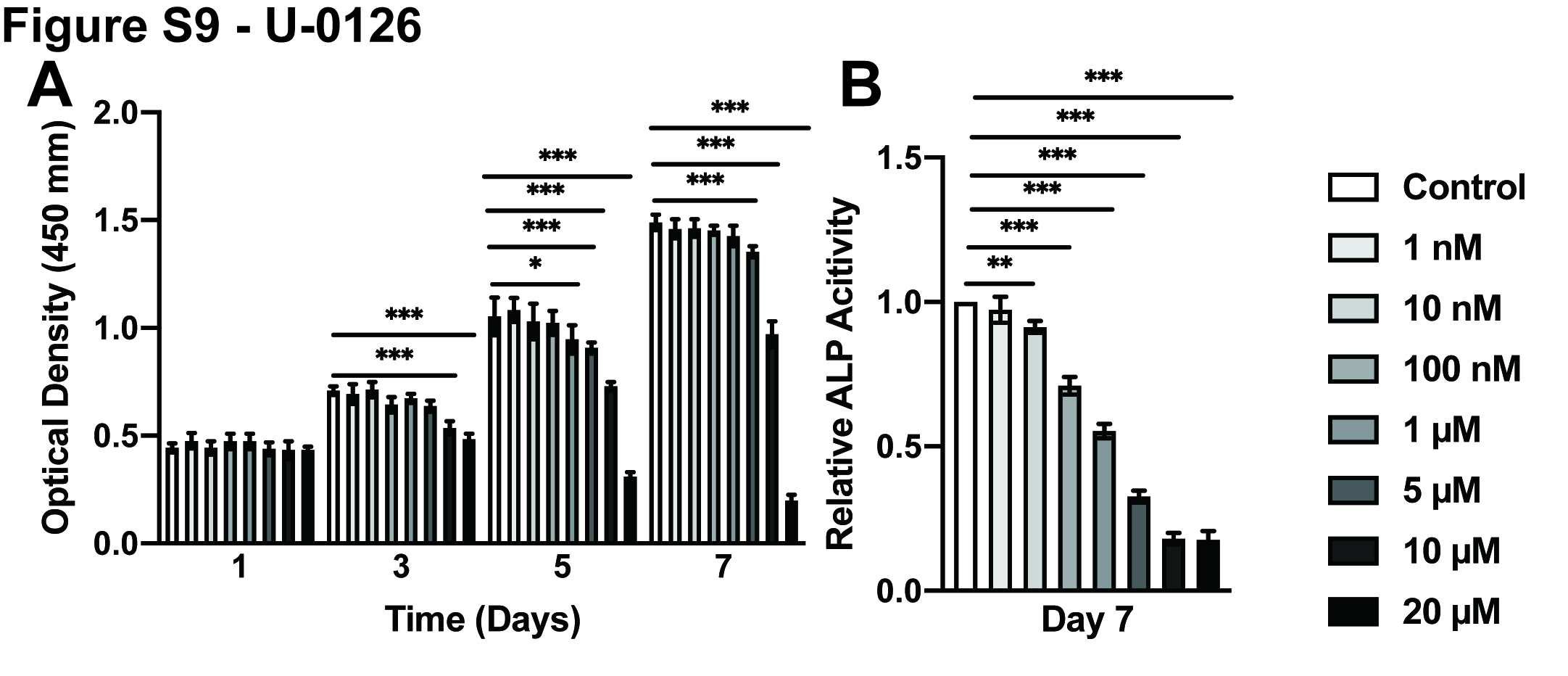

Supplement: Supplementary file 9 — Additional file 9 : Figure S9. Optimization of the concentration of U0126, a MEK1/2 inhibitor, on hBMSCs using CCK-8 and ALP activity assays. (A) CCK-8 analysis of hBMSCs treated with various concentrations (0, 1, 10, 100 nmol/L and 1, 5, 10, 20 μmol/L) of DA on days 1, 3, 5, and 7 (n = 3 for all groups). (B) ALP activity assay evaluation of hBMSCs osteogenic differentiation under the same concentration of DA on day 7 (n = 3 for all groups). Statistical significance was assessed by One-way ANOVA test; *P < 0.05; **P < 0.01; ***P < 0.001. [file 13287_2019_1529_MOESM9_ESM.tif]
